# Development of a Macrophage-Based ADCC Assay

**DOI:** 10.3390/vaccines9060660

**Published:** 2021-06-17

**Authors:** Melissa B. Uccellini, Sadaf Aslam, Sean T. H. Liu, Fahmida Alam, Adolfo García-Sastre

**Affiliations:** 1 Department of Microbiology, Icahn School of Medicine at Mount Sinai, New York, NY 10029, USA; melissa.uccellini@mssm.edu (M.B.U.); sadaf.aslam@mssm.edu (S.A.); sean.liu@mountsinai.org (S.T.H.L.); fahmida.alam@mssm.edu (F.A.); 2 Global Health and Emerging Pathogens Institute, Icahn School of Medicine at Mount Sinai, New York, NY 10029, USA; 3 Department of Medicine, Division of Infectious Diseases, Icahn School of Medicine at Mount Sinai, New York, NY 10029, USA; 4 The Tisch Cancer Institute, Icahn School of Medicine at Mount Sinai, New York, NY 10029, USA

**Keywords:** ADCC, macrophage, influenza, hemagglutinin

## Abstract

Fc-dependent effector functions are an important determinant of the in vivo potency of therapeutic antibodies. Effector function is determined by the combination of FcRs bound by the antibody and the cell expressing the relevant FcRs, leading to antibody-dependent cellular cytotoxicity (ADCC). A number of ADCC assays have been developed; however, they suffer from limitations in terms of throughput, reproducibility, and in vivo relevance. Existing assays measure NK cell-mediated ADCC activity; however, studies suggest that macrophages mediate the effector function of many antibodies in vivo. Here, we report the development of a macrophage-based ADCC assay that relies on luciferase expression in target cells as a measure of live cell number. In the presence of primary mouse macrophages and specific antibodies, loss of luciferase signal serves as a surrogate for ADCC-dependent killing. We show that the assay functions for a variety of mouse and human isotypes with a model antigen/antibody complex in agreement with the known effector function of the isotypes. We also use this assay to measure the activity of a number of influenza-specific antibodies and show that the assay correlates well with the known in vivo effector functions of these antibodies.

## 1. Introduction

Antibody function results from a combination of antigen specificity determined by the Fab region, and effector function determined by the Fc region. Fc receptors comprise a large family of cell surface receptors, including both classical FcRs and non-classical C-type lectin receptors, which are expressed on a variety of innate immune cells including macrophages, neutrophils, and NK cells. Individual Fc receptors have differing affinities, specificities for antibody isotype, and preferences for binding to monomeric Ig or immune complexes. The functional outcome of the binding of antibodies to Fc receptors results from the combination of signaling events through both inhibitory and activating receptors, which differ in their expression pattern on immune cell subsets. Signaling culminates in antibody-dependent cellular cytotoxicity (ADCC), antibody-dependent cellular phagocytosis (ADCP), and other outcomes such as cytokine production [1].

While NK cells have classically been described as effectors of ADCC, in vivo evidence suggests that macrophages play a crucial role in the effector function of a variety of antibodies [2]. Macrophage depletion by liposomal clodronate abrogates the ability of anti-CD20 antibodies to deplete B cells [3], of anti-CD142 [4] and anti-CD40 [5] antibodies to inhibit tumor growth, and of non-neutralizing anti-HA antibodies to protect from influenza virus infection [6]. In addition, treatment with the macrophage stimulating cytokine granulocyte-macrophage colony-stimulating factor (GM-CSF) enhances the efficacy of anti-CD20 and anti-GD2 antibodies for the elimination of neuroblastoma [7]. Anti-CD47 antibodies, which enhance the ability of macrophages to phagocytose target cells, also synergize with anti-CD20 [8,9]. In contrast to NK cells, which circulate in the blood and can be purified, macrophages require differentiation for a week in the presence of macrophage colony stimulating factor (M-CSF), and are hard to transfect, making them more difficult to be used in ex vivo assays to investigate the ADCC properties of antibodies.

A variety of assays exist to measure antibody effector function, which have advantages and disadvantages. Bead-based approaches can measure antibody binding and/or uptake of immune complexes into different cell types using flow cytometry or Luminex readouts [10]. However, beads may not accurately represent the density and spatial configuration of antigens on tumors or infected target cells. Cell-based approaches rely on target cells expressing the antigen of interest and a label such as green fluorescent protein (GFP) or carboxyfluorescein succinimidyl ester (CFSE) in combination with flow cytometry-based readouts [11,12], which are relatively time-intensive and lower throughput. Other cell-based approaches rely on viability readouts such as ^51^Cr release or lactate dehydrogenase (LDH) [13,14]. Macrophages take up ^51^Cr rather than releasing it into the medium, and LDH assays do not differentiate between target cell and macrophage cell death, making these approaches problematic. Another widely used assay for measuring ADCC relies on Jurkat cells expressing FcγRIIIa and an nuclear factor of activated T cells (NFAT) reporter [15]. This assay serves as a surrogate for NK-cell-mediated ADCC; however, expression of a single Fc receptor on a reporter cell line is of unknown in vivo relevance. In addition, all of these methods are endpoint assays, so do not allow for kinetic measurements in the same sample.

Given the evidence suggesting the importance of macrophages for the in vivo activity of a number of antibodies, and their expression of the full complement of Fc receptors, here, we sought to develop a macrophage-based assay to measure the ADCC activity of antibodies that could be easily adapted to high-throughput measurements. The assay relies on previously described methods for bioluminescence-based cytotoxicity assays using luciferase expression in the target cell [16] in combination with primary mouse macrophage effector cells. Upon target cell killing, ATP content is lost, and luciferase is rapidly degraded, leading to loss of luminescence. The assay relies on the passive diffusion of the D-luciferin substrate into the target cells and does not require cell lysis, allowing kinetic measurement of killing. Importantly, we show that the assay reflects the known in vivo activities of a number of antibodies and isotypes.

## 2. Materials and Methods

### 2.1. Mice

Eight- to ten-week-old female C57BL/6J mice were purchased from Jackson Labs (strain 000664). Animal studies were approved by the Institutional Animal Care and Use Committee of Icahn School of Medicine at Mount Sinai. Mice were housed in a barrier facility at the Icahn School of Medicine at Mount Sinai under specific pathogen-free conditions in individually ventilated cages and fed irradiated food and filtered water.

### 2.2. Antibodies

Trastuzumab and Rituximab antibodies were purchased from Invivogen: anti-HER2-Tra-hIgG1 (her2tra-mab1), anti-hCD20-hIgG1 (hcd20-mab1), anti-hCD20-hIgG1NQ (hcd20-mab12), anti-hCD20-hIgG2 (hcd20-mab2), anti-hCD20-hIgG3 (hcd20-mab3), anti-hCD20-IgG4 (hcd20-mab4), anti-hCD20-mIgG1 (hcd20-mab9), and anti-CD20-mIgG2a (hcd20-mab10). mIgG2b isotype control clone MPC-11 (559530) was purchased from BD. CR9114, FI6, 2B06, 2G02, 6F12, 8H9, and KB2 were kindly provided by Florian Krammer; see also Table 1.

### 2.3. Generation of Cell Lines

The gfp-luciferase fusion protein was cloned out of pAAVS1-CAG-GFPluc2 (Addgene 80493) [17] into the XhoI and XbaI sites of pLVX-IRES-Puro (Takara 632183). The chimeric hemagglutinin protein containing an H6 head and an H1 stalk (cH6/1) was cloned from pCAGGS-cH6/1 [18] into the MCS of pLVX-IRES-Neo (Takara 632181) using infusion cloning. Lentiviruses were produced using the Lenti-X Packaging Single Shots System (Takara, Mountain View, CA, USA) and concentrated using Optiprep (Sigma, St. Louis, MO, USA). Infection of Madin-Darby Canine Kidney (MDCK) cells was carried out in the presence of 8 ug/mL polybrene; infection of Rajis was conducted in the presence of 6 ug/mL DEAE-dextran on human fibronectin (Sigma) coated plates. MDCK-gfp-luc-cH6/1 cell lines were made by infecting MDCK-cH6/1 cell lines [18] with gfp-luc lentivirus; a control MDCK-gfp-luc cell line was made by infecting MDCKs with gfp-luc lentivirus; Raji-gfp-luc-cH6/1 cell lines were made by infecting Rajis with gfp-luc lentivirus followed by cH6/1 lentivirus. MDCK-gfp-luc-cH6/1 cells were selected and maintained in Dulbecco’s Modified Eagle Medium (DMEM) containing 10% fetal bovine serum (FBS) and Penicillin, Streptomycin, l-glutamine, 0.25 ug/mL puromycin, and 500 ug/mL hygromycin; Raji-gfp-luc-cH6/1 cell lines were selected and maintained in macrophage media containing 0.25 ug/mL puromycin and 500 ug/mL geneticin. Raji clones were obtained by limiting dilution.

### 2.4. Macrophage Cell Culture

Bone marrow was obtained from the femurs and tibias of mice, RBCs were lysed and cells were cultured for 7 days in RPMI 1640 (Thermo Fisher, Waltham, MA, USA) containing 10% FBS (Hyclone, GE Healthcare, Boston, MA, USA), Penicillin, Streptomycin, l-glutamine, Hepes (Cellgro, Fisher Scientific, Waltham, MA, USA), β-ME, and 10 ng/mL rmM-CSF (R&D Systems, Minneapolis, MN, USA). Macrophages were removed from the plate by scraping following incubation with cold PBS.

### 2.5. Immunofluorescence

To confirm the expression of cH6/1, MDCK and Raji cell lines were plated on human fibronectin (Sigma, St. Louis, MO, USA) coated plates, fixed in 10% formaldehyde, and blocked with blocking buffer (1% BSA/PBS). Cells were incubated with 30 ug/mL 8H9 in blocking buffer for 1 h at room temp, washed, and incubated with 2 ug/mL anti-mouse IgG-Alexa 555 (Invitrogen, Waltham, MA, USA) and DAPI in blocking buffer for 1 h at room temp, followed by washing and imaging with LSM888.

### 2.6. Flow Cytometry

MDCK and Raji cell lines in FACs buffer (3% FBS/2 mM EDTA/PBS) were incubated with 30 ug/mL of the indicated antibodies for 1 h at RT with shaking, washed 2× with FACs buffer, and incubated with goat anti-mouse Ig-APC (BD 550826) or mouse anti-human IgG-APC (BD 550931) for 30 min at 37 °C, washed 2× with FACs buffer, and fixed in 2% PFA. Cells were run on a BD FACSCaliber and analyzed with FlowJo.

### 2.7. ADCC Assay

MDCK and Raji target cells were plated in 50 uL of macrophage media at 0.6 × 10^6^/mL in white 96-well flat-bottom tissue-culture treated plates (Corning 3917, Corning, NY, USA), followed by the addition of 50 uL of beetle luciferin (Promega, Madison, WI) at 600 ug/mL, and macrophages at 1.2 × 10^6^/mL (for 2:1 ratio) or 1.8 × 10^6^/mL (for 3:1 ratio). Cells were incubated for 30 min at 37 °C and an initial zero-time luminescence reading was taken, followed by the addition of 50 uL antibodies at 4 ug/mL or 0.4 ug/mL. The plate was removed from the incubator at indicated time points and equilibrated to room temperature for 5 min, followed by reading luminescence on a BioTek plate (Winooski, VT) reader with a 1 s integration time. Kinetic measurements represent the same well sampled at different time points after a single addition of beetle luciferin. As there was some variation in luciferase signal at different time points, and with the addition of macrophages, data were normalized to the mock sample either + or − macrophages at each individual time point. For example: (Raji 5 h)/(Mock 5 h)*100 or (Raji + MAC 5 h)/(Mock + MAC 5 h)*100. Statistical significance was calculated by 2-way ANOVA in GraphPad Prism (San Diego, CA, USA).

## 3. Results

### 3.1. Development of a Macrophage-Based ADCC Assay

**Figure 1:**
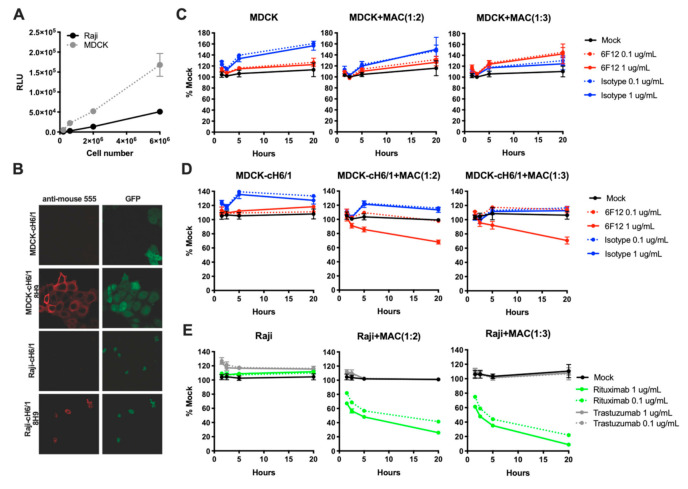
Macrophage-based ADCC assay. (**A**) Increasing numbers of MDCK or Raji target cells were incubated with D-luciferin substrate and luminescence was measured at 1 h post-addition. (**B**) MDCK-cH6/1 and Raji-cH6/1 cells were stained with HA-specific antibody 8H9 followed by anti-mouse Alexa 555. (**C**) MDCK target cells were incubated with 6F12 or isotype control Ab in the absence or presence of various ratios of macrophages and luminescence was measured at the indicated time points. No killing was observed in the absence of antigen expression. (**D**) MDCK-cH6/1 target cells were incubated as in C. Killing was observed in the presence of antigen expression and the specific antibody. (**E**) Raji target cells were incubated with the indicated antibodies. Killing was again observed with the specific antibody.

In order to develop a macrophage-based ADCC assay, we first established target cell lines expressing a GFP-luciferase fusion protein. To validate that luminescence serves as a correlate of live cell number, we plated increasing concentrations of cells and measured luminescence. As shown in Figure 1A, live cell number correlates with luminescence. In order to develop an assay for measuring the ADCC activity of influenza HA-specific antibodies, we then generated GFP-luc cell lines co-expressing the cH6/1 chimeric influenza virus HA protein. Because humans do not have antibody titers against H6 hemagglutinin, this chimeric construct allows measurement of stalk-specific antibodies. We confirmed expression of the chimeric protein by immunofluorescence (Figure 1B). We next tested the ability of the target cell lines to be killed by primary mouse macrophages in combination with HA-specific or control antibodies. MDCK cells lacking expression of the cH6/1 antigen were not killed in the presence of the HA-specific antibody 6F12 or isotype control antibody (Figure 1C). In contrast, loss of luciferase signal was observed using MDCK target cells expressing cH6/1 at higher doses of 6F12, but not isotype control antibody (Figure 1D), indicating specific killing in the presence of the antigen/antibody combination. As an additional proof of concept, we also incubated Raji target cells with Rituximab, which binds to the CD20 antigen expressed on Raji cells or the negative control antibody Trastuzumab. Specific killing was seen with both high and low doses of Rituximab, but not Trastuzumab (Figure 1E).

### 3.2. ADCC Activity of a Panel of HA-Specific Antibodies

**Figure 2:**
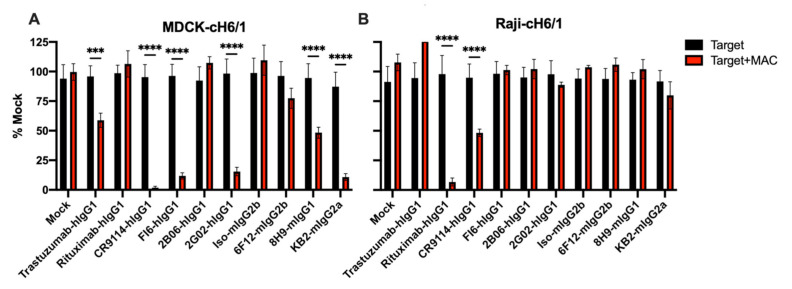
ADCC activity of a panel of HA-specific antibodies. (**A**) MCDK-cH6/1 target cells were incubated with the indicated antibodies at 1 ug/mL in the presence or absence of macrophages at a 1:3 ratio. ADCC activity was measured at 24 h. A number of antibodies showed ADCC activity on MDCK-cH6/1 target cells. (**B**) Raji-cH6/1 target cells were incubated as in A. Killing was only observed with CR9114 and the control antibody Rituximab. *** *p* < 0.001, **** *p* < 0.0001.

Much more efficient and complete killing was observed with Raji-cH6/1 target cells in combination with Rituximab than with MDCK-cH6/1 target cells. MDCK cells form a tight monolayer, while Raji cells grow in suspension. We reasoned that this may allow macrophages to kill Raji cells more efficiently, although a number of other factors, including target antigen density, antibody specificity, or antibody isotype, could also account for the observed differences. We therefore tested both MDCK-cH6/1 and Raji-cH6/1 target cells for ADCC activity against a panel of HA-specific antibodies (Table 1). Using MDCK-cH6/1 target cells, a number of antibodies including CR9114, FI6, 2G02, and KB2 showed complete killing. Other antibodies, including 6F12 and 8H9, showed partial killing (Figure 2A). In contrast, using Raji-cH6/1 target cells, only CR9114 showed partial killing (Figure 2B), despite the control antibody Rituximab showing complete killing. The reason for the differential target cell killing is unknown. We observed ADCC activity of both head and stalk-reactive antibodies, consistent with reports suggesting Fc receptors are required for the in vivo activity of both types of antibodies, although the absolute requirements may be different [19,20].

### 3.3. Antibody Binding

**Figure 3:**
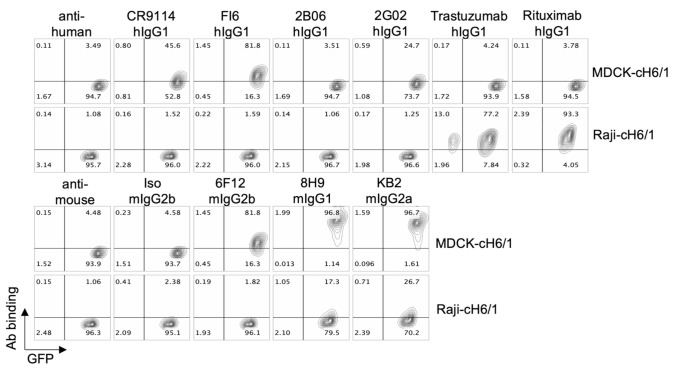
Binding of a panel of HA-specific antibodies. MDCK-cH6/1 and Raji-cH6/1 cells were incubated with the indicated antibodies followed by either human (**top**) or mouse (**bottom**) secondary antibody. GFP expression and cell surface binding were measured by flow cytometry.

In order to determine if differences in antigen levels or antibody binding explained the differential killing of MDCK and Raji target cells, we incubated the target cell lines with the same panel of HA-specific antibodies and measured binding to the cell surface by flow cytometry. Both cell lines expressed the cH6/1 antigen as indicated by the binding of the 8H9 and KB2 antibodies (Figure 3). However, Raji-cH6/1 cells had a much lower mean fluorescence intensity (MFI) compared to MDCK-cH6/1 cells, indicating less surface HA expression. While all antibodies that bound to MDCK-cH6/1 cells induced killing, the level of killing did not correlate with the level of surface binding. Surprisingly, only the 8H9 and KB2 antibodies, which showed the highest binding to MDCK-cH6/1 cells, bound to Raji-cH6/1 cells. It is possible that higher antigen density is required for the other antibodies to bind to Raji-cH6/1 cells, or that other factors such as sialic acid interactions may influence differential binding between the two cell types. Regardless, the data demonstrate that target cell selection is critical for ADCC assay development.

### 3.4. ADCC Activity of Human and Mouse Isotypes

**Figure 4:**
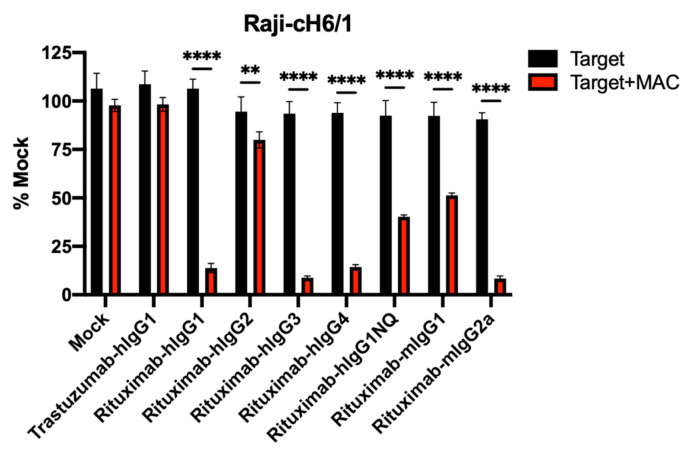
ADCC activity of mouse and human Rituximab isotypes. Raji-cH6/1 target cells were incubated with the indicated antibodies at 1 ug/mL in the presence or absence of macrophages at a 1:3 ratio. ADCC activity was measured at 24 h. ** *p* < 0.01, **** *p* < 0.0001.

The panel of HA-specific antibodies tested contained a variety of different mouse and human isotypes. Antibody isotype is known to influence ADCC activity. In order to test the isotype’s impact in our assay, as well as the ability of both mouse and human isotypes to function in the assay with mouse macrophages, we tested a variety of mouse and human Rituximab isotypes in the assay. The human IgG1, IgG3, and IgG4 antibodies induced complete killing of Raji-cH6/1 target cells, while the IgG2 antibody did not induce killing (Figure 4). The human IgG1NQ antibody, which removes the N297 glycosylation site in the constant region, displayed reduced activity as expected. The mouse IgG2a antibody displayed complete killing, while the IgG1 isotype induced partial killing.

## 4. Discussion

Here, we report the development of an ADCC assay based on primary mouse macrophages. The assay shows the activity of one antibody (CR9114) on both MDCK and Raji target cells; however, the activity of a number of other antibodies is only evident on MDCK target cells. A number of factors have been shown to influence ADCC activity, including antibody affinity, antigen expression level [21], and isotype [3,22]. Antibodies with higher affinity have been reported to be less dependent on antigen expression levels [21]; therefore, it is possible that CR9114 has a higher affinity than the other antibodies for the cH6/1 antigen. Raji target cells also expressed lower levels of HA, as indicated by the lower binding of 8H9 and KB2 by flow cytometry. Interestingly, we were able to observe partial ADCC activity with CR9114, despite no measurable binding to the cell surface of Raji cells, suggesting that the staining conditions may not accurately reflect the ADCC assay conditions when macrophages are present. It is possible that the binding of the other antibodies requires lower avidity interactions that cannot occur in the context of the lower antigen density on Raji cells. Additionally, ADCC activity of anti-HA antibodies has been reported to be abrogated in the absence of sialic acid interactions between the HA receptor binding domain and sialic acids on target cells [23]. Therefore, it is possible that sialic acid expression on MDCK cells is optimal compared to Raji cells. Regardless, it is clear that target cell selection and antigen expression level are key factors for ADCC.

Antibody isotype also plays a key role in ADCC activity. In our studies, hIgG1 antibodies, which bound at low (CR9114 and 2G02), intermediate (FI6), and high (Rituximab-hIgG1) levels, all showed complete ADCC activity in the assay conditions. This suggests that ADCC activity may be more dependent on antibody isotype than level of binding, although differences may be revealed at lower doses or earlier time points. mIgG2a antibodies are generally considered the functional equivalent of hIgG1 due to high FcγR binding. In agreement with this, our mIgG2a antibodies, which bound at high levels (KB2 and Rituximab-mIgG2a), both showed complete ADCC activity. Rituximab antibodies of the hIgG3 and hIgG4 subclasses also showed complete ADCC activity, suggesting that in the case of an optimal antibody, isotype may be less important. In agreement with their less efficient FcγR binding profile, mIgG1 antibodies that bound at high levels (8H9 and Rituximab-mIgG1) both showed only partial ADCC activity, as did the intermediate binder 6F12 of the mIgG2b isotype. These data are in agreement with ADCC studies using human hapten antibodies, which showed activity with both hIgG1 and hIgG3 with human PBMCs [22], and mouse anti-CD20 studies, which showed in vivo activity was most efficient with mIgG2a, followed by mIgG1 and mIgG2b [3]. This is in contrast to the commercial FcγRIIIa reporter assay for NK cells, which works with hIgG1, but not hIgG4 [15]. Importantly, our ADCC results are in agreement with the in vivo FcR dependence of FI6, 2G02 and 6F12 [19]. 6F12 was demonstrated to be FcR-dependent and alveolar macrophage-dependent in a dose-dependent manner in vivo [6], which may explain its partial ADCC activity in our assay.

Primary mouse macrophages are not an ideal effector cell for a high-throughput assay and are not amenable to traditional immortalization methods. We were unable to observe specific ADCC activity with a variety of transformed mouse and human macrophage cell lines including RAW or J774 mouse cell lines, or K562, THP1, or U937 human cell lines. In addition, other methods of immortalization, including Hoxb8 [24] and J2 [25], did not result in ADCC activity (not shown). While we could observe the ADCC activity of human antibodies in our assay, ideally, the assay should be adapted for use with human PBMCs due to differences in mouse and human FcRs. Alternatively, the assay could be used to identify genes that serve as reliable reporters for macrophage ADCC activation, in order to develop a macrophage assay similar to the commercial FcγRIIIa NFAT reporter assay for NK cell ADCC.

## Figures and Tables

**Table 1:** Antibodies used in this study.

Antibody	Specificity	Isotype	Source/Reference
Trastuzumab	HER2	hIgG1	Invivogen
Rituximab	CD20	hIgG1	Invivogen
Rituximab	CD20	hIgG2	Invivogen
Rituximab	CD20	hIgG3	Invivogen
Rituximab	CD20	hIgG4	Invivogen
Rituximab	CD20	hIgG1NQ	Invivogen
Rituximab	CD20	mIgG1	Invivogen
Rituximab	CD20	mIgG2a	Invivogen
CR9114	Flu A and B stalk	hIgG1	PMID 22878502
FI6	Flu A Group 1 and 2 HA stalk	hIgG1	PMID 21798894
2B06	Flu A Group 1 and 2 HA stalk	hIgG1	PMID 25689254
2G02	Flu A Group 1 and 2 HA stalk	hIgG1	PMID 22615367
Iso	unknown	mIgG2b	BD clone MPC-11
6F12	Flu H1 stalk	mIgG2b	PMID 22491456
8H9	Flu H6 head	mIgG1	PMID 26512088
KB2	Flu H1 stalk	mIgG2a	PMID 22398287

## Data Availability

Not applicable.
